# The Impact of Government Subsidies on the Low-Carbon Supply Chain Based on Carbon Emission Reduction Level

**DOI:** 10.3390/ijerph18147603

**Published:** 2021-07-16

**Authors:** Biao Li, Yong Geng, Xiqiang Xia, Dan Qiao

**Affiliations:** 1 College of Business, Zhengzhou University, Zhengzhou 450001, China; lib0023@zzu.edu.cn (B.L.); qiao0205@126.com (D.Q.); 2 College of Environmental Science and Engineering, Shanghai Jiao Tong University, Shanghai 200240, China; ygeng@sjtu.edu.cn

**Keywords:** LCSC, government subsidies, carbon emission reduction level, game theory model

## Abstract

To improve low-carbon technology, the government has shifted its strategy from subsidizing low-carbon products (LCP) to low-carbon technology. To analyze the impact of government subsidies based on carbon emission reduction levels on different entities in the low-carbon supply chain (LCSC), game theory is used to model the provision of government subsidies to low-carbon enterprises and retailers. The main findings of the paper are that a government subsidy strategy based on carbon emission reduction levels can effectively drive low-carbon enterprises to further reduce the carbon emissions. The government’s choice of subsidy has the same effect on the LCP retail price per unit, the sales volume, and the revenue of low-carbon products per unit. When the government subsidizes the retailer, the low-carbon product wholesale price per unit is the highest. That is, low-carbon enterprises use up part of the government subsidies by increasing the wholesale price of low-carbon products. The retail price of low-carbon products per unit is lower than the retail price of low-carbon products in the context of decentralized decision making, but the sales volume and revenue of low-carbon products are greater in the centralized decision-making. The cost–benefit-sharing contract could enable the decentralized decision model to achieve the same level of profit as the centralized decision model.

## 1. Introduction

The low-carbon economy is the future of sustainable development, and carbon emission reduction has become a common goal [1,2,3]. The development of a low-carbon economy requires a shift to a green and sustainable growth model and reduction of continuing threats to natural ecosystems and energy security [4,5,6]. The development of a low-carbon economy relies on the adjustment of industrial, energy, and consumption structures and requires policy support [7,8,9,10]. In China, Ghana, Australia and the United States, high-energy-consuming and carbon-intensive industries such as electricity are the main factors contributing to the increase in carbon emissions [11]. Countries around the world have adopted measures to reduce greenhouse gas emissions, such as the United Nations Framework Convention on Climate Change, the Kyoto Protocol, and the Copenhagen Protocol, which emphasize the need for countries to work together to ensure sustainable economic development. In 2019, a total of 20 carbon emissions trading systems were in operation [12]. The global carbon market covers about 8% of total greenhouse gas emissions, and the total GDP of the regions accounts for about 37% of global GDP. Carbon emissions trading has become an effective ecological governance tool based on the principles of internationalization. For example, South Korea enacted the Framework Act on Low Carbon Green Growth in 2010, implementing complementary policies such as energy structural transformation and energy conservation into law [13,14]. Since 2010, the focus of German carbon emission reduction policies has shifted from niche technology development to the destabilization of the existing high-carbon regime [15]. In recent years, China’s share of the world’s total carbon emissions has been high [16,17,18]. In 2011, the Chinese government issued the Notice on the Work of Piloting Carbon Emissions, approving seven provinces and municipalities to carry out pilot carbon emissions trading programs and promoting the construction of a nationally unified carbon market [19]. In 2017, China’s National Development and Reform Commission (NDRC) released the National Emissions Trading Market Construction Plan (Power Generation Sector), officially launching the national carbon emissions trading system, which may be the largest emissions trading platform in the world [20,21,22,23,24]. In 2020, during the 75th United Nations General Assembly, the Chinese government pledged that the country would strive to achieve carbon neutrality before 2060. These measures and policies indicate that the low-carbon development model is widely adopted for future economic development [25,26,27,28,29].

Subsidy policy plays a critical role in renewable energy development because environmental efficiencies of subsidies decrease with the subsidy degree [30]. To reduce carbon emissions, governments subsidize low-carbon enterprises or consumers that contribute to a low-carbon economy. Governments also aim to improve low-carbon enterprises’ research and development (R&D) level [31], remanufacturing activities [32,33], consumer preferences and behavior [34,35,36]. At present, the main policy measures are increasing carbon taxes and implementing carbon emission reduction subsidies. Scholars generally believe that subsidy policies are more effective than carbon tax policies in curbing carbon emissions. This may be because, although remanufacturing subsidies promote the profit of firms, carbon regulation hurts profits [37,38]. On the one hand, government subsidies can increase the remanufacturing activities and the profits of low-carbon companies. Government subsidies stimulate the demand for energy performance contracting and the profit of energy service companies [39]. On the other hand, government subsidies can encourage low-carbon enterprises in the supply chain to invest in carbon emission reduction [40]. The energy sector’s access to technology subsidies is conducive to a reduction in carbon emissions but will not affect economic growth [19]. Sharing costs is an effective way to promote cooperation between retailers and low-carbon companies to achieve carbon emission reduction [41]. The governments set appropriate subsidy levels to encourage low-carbon enterprise to adopt desired channel structures [42]. For example, when the climate change levy system was introduced in the UK in 2001, it raised £1.2 billion a year, of which £100 million went to subsidies to promote a low-carbon economy. In the UK, due to the implementation of public policies such as carbon emission reduction subsidies, primary energy consumption fell from 152.3 in 2007 to 139.8 in 2009, resulting in a reduction of about 8% in carbon emissions during the same period. Since then, the same downward trend has also appeared in 2010–2015 [43]. Since 2007, Canada has implemented a subsidy program that gives 1000–2000 Canadian dollars to consumers for each renewable energy vehicle. Denmark has adopted a policy that provides financial incentives for biomass power generation. The US government announced that 20–30% of the cost of equipment for companies producing low-carbon products could be used for tax deductions, and relevant low-carbon companies and individuals could also enjoy tax reductions ranging from 10% to 40%. In 17 counties in Tennessee and one county in Kentucky, the annual county-level cost of using tax-based subsidies to provide forest carbon sequestration is between $15.56 and $563.58 per carbon ton, and this method effectively reduces the risk of deforestation and is conducive to carbon reduction [44]. The Chinese government formulated the Interim Measures for the Administration of Low-Carbon Products Certification in 2013. Since 2018, the Chinese government has invested in large subsidies for new energy fields, such as hydrogen vehicles, energy-saving products, and eco-friendly technologies and equipment, in order to improve the core competitiveness of its industrial chain.

However, long-term undifferentiated government subsidies for low-carbon industries may impose fiscal pressure and reduce market allocation efficiency. When a subsidy policy was superior to a carbon tax policy, social welfare and economic benefits were improved [45]. Therefore, scholars have proposed optimization strategies for combining tax policies and catering to consumers’ low-carbon preferences [46,47]. When government subsidies are combined with a carbon tax, an effective combination of economic growth and carbon emission reduction can be achieved [48]. Government subsidies fully account for industry characteristics and the energy efficiency levels of various industries [39]. Government subsidies can increase the profits of supply chain entities while reducing carbon emissions throughout the supply chain, but government carbon subsidies should be within reasonable limits [49]. In addition, government subsidies should also account for differences in consumer awareness of carbon emissions [50]. Therefore, the Chinese government has gradually changed its subsidy strategy, shifting to subsidies based on carbon emission reduction levels. Under the new strategy, higher carbon emission reduction levels and associated costs gain larger government subsidies [51,52]. Thus it is important to study the impact of carbon emission reduction level-based government subsidies on the LCSC, as research can support government decision making in refining its low-carbon subsidy policy and ultimately promote the development of LCPs.

Both carbon tax policies and government subsidies will significantly affect the development of the LCSC. However, due to the low overall level of low-carbon industry development, the current government’s leading policy still relies on government subsidies [10]. In contrast to previous research that focused on suppliers and low-carbon enterprises within the supply chain [26,40,53], this paper presents a game theory model consisting of a low-carbon enterprise and a retailer. According to the methodology of various studies [54,55,56,57], this paper compares how the government’s choice of different subsidy recipients affects the LCSC and then analyzes the impacts of decentralized and centralized decision-making systems on the supply chain. Finally, we provide an LCSC coordination mechanism under the decentralized decision-making system. The aim of this research is to provide support to the government in improving its subsidy policy and increasing the cooperation of LCSC participants. Based on existing research, this paper intends to address the following three issues:

(1) Whether and how different subsidy recipients produce different effects on the LCSC;
(2) The impact of government subsidies under decentralized and centralized decision making on the LCSC;
(3) How LCSC coordination can be implemented under decentralized decision making based on the cost–benefit-sharing contract in order to achieve benefits compared to benefits under centralized decision making.

The structure of this paper is as follows. Section 2 briefly presents the game theory model. Section 3 explains the process of model construction, in which we show how the government’s different choices of subsidy recipients based on carbon emission reduction levels affect the LCSC. Section 4 uses electric vehicles as an example to perform mathematical analysis and draw some inferences. Section 5 discusses the research proposition, management insights, and research outlook.

## 2. Preparations before Modeling

### 2.1. Problem Statements

**Figure 1:**
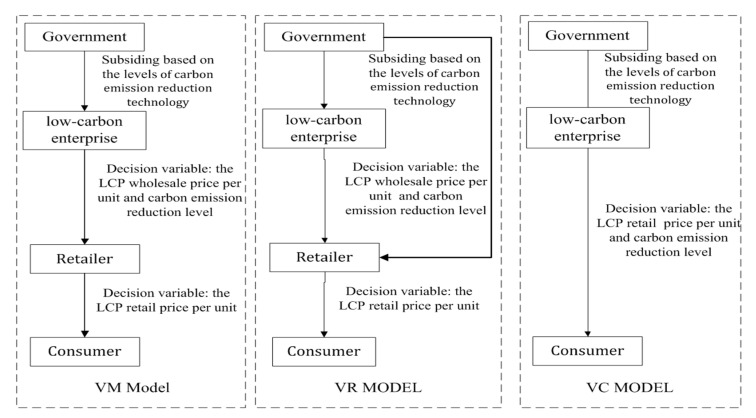
Flowchart of the game model.

To promote carbon emission reduction technologies, the government subsidizes the LCSC based on levels of the carbon emission reduction technology. Subsidies for sources with low emissions to energy price ratios can change the relative price of low and high emissions energy sources and increase welfare benefits [58]. At present, China and other countries have gradually changed the carbon emission subsidy model, and determined the amount of subsidy by identifying carbon emission levels. This is an effective attempt to improve resource utilization efficiency and reduce carbon emissions. An example is Guangzhou’s subsidy strategy for electric vehicles: initially, fuel cell vehicles were given local subsidies at a ratio of no more than 1:1 of the national subsidy standard. For purely electric vehicles, local subsidies were given at a ratio of no more than 1:0.5 of the national subsidy standard. For plug-in hybrid (including supercharged) vehicles, local subsidies were provided at a ratio of no more than 1:0.3 of the national subsidy standard. After 2020, the subsidy policy will switch to a decreasing differential subsidy for vehicles with an electric range of no less than 400 km and a range of 250~400 km. The game theory model between a low-carbon enterprise and a retailer is established for analysis of the effect of government subsidies and the impact of subsidizing different recipients on the LCSC. In the decentralized decision model, the decision variables of the low-carbon enterprise are the wholesale price per unit and the levels of carbon emission reduction technology. Second, the decision variable of the retailer is the LCP retail price per unit in view of the wholesale price per unit and the levels of carbon emission reduction technology. By contrast, in the situations of centralized decision making, low-carbon companies are also responsible for production and sales, so the decision variable is the LCP retail price per unit and the levels of carbon emission reduction technology. The decision-making modes are shown in Figure 1.

### 2.2. Notation Description

The model notation used in this paper is described in Table 1.

### 2.3. Model Demand Function

This study uses a classical demand function [10], and the relationship between demand and the LCP retail price per unit is as follows:

$$q_{i}=Q-\alpha p_{i}\;,\;\mathrm{where}\;i\in \left\{VM,VR,VC\right\}$$

The cost of carbon emission reductions is a concave quadratic function. The cost is $\frac{k\tau_{i}^{2}}{2}$, where i∈{VM,VR,VC}.

## 3. Model

### 3.1. Model Development

To make this study meaningful, in situations of decentralized decision making, it is assumed that $4k-\alpha v^{2}>0$; in situations of centralized decision making, it is assumed that $2k-\alpha v^{2}>0$. Otherwise, entities in the LCSC will not choose to reduce carbon emission.

In situations of decentralized decision making, the government subsidizes the low-carbon enterprise:

(1) $$\pi_{VMM}=\left(w_{VM}-c+\tau_{VM}v\right)q_{VM}-\frac{k\tau_{VM}^{2}}{2}$$

(2) $$\pi_{VMR}=\left(p_{VM}-w_{VM}\right)q_{VM}$$

The government can also subsidize the retailer:

(3) $$\pi_{VRM}=\left(w_{VR}-c\right)q_{VR}-\frac{k\tau_{VR}^{2}}{2}$$

(4) $$\pi_{VRR}=(p_{VR}-w_{VR}+\tau_{VR}v)q_{VR}$$

In situations of centralized decision making:

(5) $$\pi_{VC}=\left(p_{VC}-c+\tau_{VC}v\right)q_{VC}-\frac{k\tau_{VC}^{2}}{2}$$

**Lemma** **1.** (i) Equation (2) with respect to $p_{VM}$ is a concave function. The optimal solution $\overset{*}{p_{VM}}$ is obtained by substituting Equation (2) into Equation (1). Equation (1) with respect to $w_{VM},\;\tau_{VM}$ is a concave function. (ii) Equation (4) with respect to $p_{VR}$ is a concave function. The optimal solution $\overset{*}{p_{VR}}$ is obtained by substituting Equation (4) into Equation (3). Equation (3) with respect to $w_{VR},\tau_{VR}$ is a concave function. (iii) Equation (5) with respect to $p_{VC},\;\tau_{VC}$ is a concave function. Please refer to Appendix A for the specific certification process.

**Proposition** **1.** *We calculated the optimal solution for the government based on carbon emission reduction levels. See Table 2.*

**Proposition** **2.** *In the decentralized decision model, the government’s choice of different subsidy recipients affects only the LCP wholesale price per unit and not the LCP retail price per unit, sales volume or profits.*

**Proposition** **3.** In the decentralized decision model, the impact of the government’s choice of different subsidy recipients on the LCP wholesale price per unit is $\overset{*}{w_{VM}}<{\overset{*}{w}}_{VR}$. Please refer to Appendix A for the specific certification process.

Similar to [59], Propositions 2 and 3 show that when governments choose to grant subsidies to the low-carbon enterprise based on the number of products sold, the low-carbon enterprise is incentivized to reduce the LCP wholesale price per unit in order to increase sales and acquire larger government subsidies. The reduction in the wholesale price per unit then leads to a decrease in the retail price per unit. On the other hand, when the government grants subsidies to the retailer, the low-carbon enterprise can acquire a part of the government subsidy by increasing the LCP wholesale price per unit. However, the impact on the LCP retail price per unit is the same regardless of whether low-carbon enterprises or retailers receive government subsidies. When the government subsidizes the low-carbon enterprise, the decrease in the LCP wholesale price per unit is greater than the decrease in the retail price per unit obtained when the government subsidizes the retailer, but the final LCP retail price per unit is set in line with the government subsidies granted to the retailer. In contrast to [59], Propositions 2 and 3 further indicate that low-carbon enterprises and retailers both obtain government subsidies through the LCP wholesale price per unit. When the government grants subsidies to the low-carbon enterprise, the retailer receives a part of government subsidies through the lowered wholesale price per unit of the low-carbon product. When the government grants subsidies to the retailer, the low-carbon enterprise receives part of government subsidies by increasing the LCP wholesale price per unit.

**Proposition** **4.** *The influences of government subsidies on the LCP wholesale price per unit, LCP retail price per unit, and sales volume are as follows:*

*(i)*  $\frac{\partial w_{VM}^{*}}{\partial v}<0,\;\frac{\partial w_{VR}^{*}}{\partial v}>0$;
*(ii)*  $\frac{\partial p_{VM}^{*}}{\partial v}=\frac{\partial p_{VR}^{*}}{\partial v}<0,\;\frac{\partial p_{VC}^{*}}{\partial v}<0$;
*(iii)*  $\frac{\partial q_{VM}^{*}}{\partial v}=\frac{\partial q_{VR}^{*}}{\partial v}>0,\;\frac{\partial q_{VC}^{*}}{\partial v}>0$.

Please refer to Appendix A for the specific certification process.

Proposition 4 shows that the LCP retail price per unit is negatively correlated with subsidies granted by the government. However, sales volume is positively correlated with government subsidies. The entity profit in the supply chain benefits from government subsidies, this conclusion is similar to [49]. When the government grants subsidies to the low-carbon enterprise, the LCP wholesale price is negatively correlated with government subsidies. The main reason is as follows: the government subsidies depend on the LCP sales volume; That is, the more LCPs that are sold, the more government subsidies they receive. This is because subsidizing the low-carbon enterprise will incentivize it to reduce the LCP wholesale price per unit, which leads to a decrease in the LCP retail price per unit and an increase in the sales volume of products [60]. In contrast to the findings in [59,60], we found that when the government subsidizes the retailer, the low-carbon enterprise increases the wholesale price per unit to indirectly obtain a part of the subsidy from the retailer, but the retail price per unit ultimately decreases because government subsidies are provided directly to the retailer. The effect of the retailer subsidy on carbon emission reductions is the same as that when the government subsidizes the low-carbon enterprise.

**Proposition** **5.** *The influences of government subsidies on the carbon emission reduction effort per unit are as follows:*

$$\frac{\partial \tau_{VM}^{*}}{\partial v}=\frac{\partial \tau_{VR}^{*}}{\partial v}>0,\;\frac{\partial \tau_{VC}^{*}}{\partial v}>0$$

Please refer to Appendix A for the specific certification process.

In contrast to the current literature, Proposition 5 shows that the carbon emission reduction effort per unit is positively related to the amount of government subsidies. This is mainly because government subsidies cover part of the costs of carbon emission reductions. To obtain more subsidies, the low-carbon enterprise aims to reduce the carbon emissions per unit of product. Furthermore, subsidizing either low-carbon enterprises or retailers has the same effect on carbon emission reduction levels.

**Proposition** **6.** *The influences of government subsidies on profit are as follows:*

*(i)*  $\frac{\partial \pi_{VMM}^{*}}{\partial v}=\frac{\partial \pi_{VRM}^{*}}{\partial v}>0$;
*(ii)*  $\frac{\partial \pi_{VMR}^{*}}{\partial v}=\frac{\partial \pi_{VRR}^{*}}{\partial v}>0$;
*(iii)*  $\frac{\partial \pi_{VC}^{*}}{\partial v}>0$.

The carbon subsidy of government could increase the profits of agents of the supply chain and deduce the carbon emission of the whole supply chain simultaneously [49]. Similar to [49], Proposition 6 shows that both low-carbon enterprise and retailer profits are positively correlated with the amount of government subsidies, and different government subsidy strategies have the same effect on the profits of low-carbon enterprises and retailers. In contrast to [49], combined with Proposition 4 and Proposition 5, we argue that, although the LCP wholesale price per unit is lower when the government subsidizes the low-carbon enterprise than that when the government subsidizes the retailer, the LCP retail price per unit remains the same in both instances, which leads to the same sales volume. Further analysis shows that, when the government grants subsidies to the low-carbon enterprise, the low-carbon enterprise will choose to reduce the LCP wholesale price per unit. When the government grants subsidies to the retailer, the low-carbon enterprise increases the LCP wholesale price per unit to acquire part of the subsidy, which maintains the same revenue per unit of product for the low-carbon enterprise and the retailer. Additionally, different government subsidy options produce the same effect on the sales volume of low-carbon products and on effort levels to reduce carbon emissions, ultimately resulting in equal benefits for the low-carbon enterprise and the retailer.

**Proposition** **7.** *The impacts of centralized or decentralized decision making on the LCP retail price per unit, sales volume, and efforts to reduce carbon emissions are as follows:*

*(i)*  $p_{VM}^{*}=p_{VR}^{*}>p_{VC}^{*}$;
*(ii)*  $q_{VM}^{*}=q_{VR}^{*}<q_{VC}^{*}$;
*(iii)*  $\tau_{VM}^{*}=\tau_{VR}^{*}<\tau_{VC}^{*}$.

Please refer to Appendix A for the specific certification process.

Similar to [54], Proposition 7 shows that under the condition of decentralized decision making, different recipients receiving subsidies produce the same effect on the LCP retail price per unit, demand, and efforts to reduce carbon emissions in the LCP. Compared to centralized decision making, however, the LCP retail price per unit, demand, and efforts to reduce carbon emissions are higher in situations of decentralized decision making. The foremost reason is that in situations of decentralized decision making, the marginal effect can be avoided [56], which reduces the cost of the LCSC. When low-carbon enterprises receive subsidies directly from the government, they reduce the LCP wholesale price per unit to promote sales, which causes a decrease in the LCP retail price per unit. In contrast to [54,56], we argue that when the retailer is the government subsidy recipient, the low-carbon enterprise increases the LCP wholesale price per unit to receive part of the subsidies from the retailer. Therefore, subsidizing retailers produces the same effect as subsidizing low-carbon enterprises on the LCP wholesale price per unit. In addition to the retail price per unit, centralized decision making also reduces the operating cost of the LCSC. Furthermore, low-carbon enterprises tend to use the saved operating costs to further reduce carbon emissions to receive even more subsidies.

To demonstrate the impact of decentralized/centralized decision making on profit, we have:

$$\pi_{VM}^{*}=\pi_{VR}^{*}=\pi_{VMM}^{*}+\pi_{VMR}^{*}=\pi_{VRM}^{*}+\pi_{VRR}^{*}=\frac{k{\left(Q-\alpha c\right)}^{2}\left(6k-\alpha v^{2}\right)}{2\alpha {\left(4k-\alpha v^{2}\right)}^{2}}$$

**Proposition** **8.** *The impact of decentralized/centralized decision making on profit is:*

$${\overset{*}{\pi }}_{VM}={\overset{*}{\pi }}_{VR}<{\overset{*}{\pi }}_{VC}$$

Please refer to Appendix A for the specific certification process.

Proposition 8 shows that when government subsidies are based on carbon emission reduction levels, low-carbon enterprise and retailer revenue in situations of centralized decision making is higher than in situations of decentralized decision making. The reason is that decentralized decision-making will produce marginal effects, which will reduce the profits of low-carbon companies and retailers. Each member’s decision making is for their own profit maximization, leading to the loss of marginal benefit [50]. By contrast, the centralized decision-making model avoids marginal effects and results in higher total revenue. As an aside, the effect of subsidies under decentralized decision making does not differ between government subsidization of retailers and that of low-carbon enterprises. In contrast to [50], Proposition 8 also highlights the need for further research into LCSC coordination mechanisms to increase the total profit of the LCSC when decisions are decentralized. The LCSC coordination mechanism is analyzed below in the form of a cost–benefit-sharing contract.

### 3.2. Cost–Benefit-Sharing Contract

When the government subsidizes the low-carbon enterprise, cost-sharing means that the low-carbon enterprise sells products to the retailer at a lower LCP wholesale price per unit [41], which is $\bar{w_{VM}}=c-\bar{\tau_{VM}v}$. At the same time, the retailer needs to bear a proportionate share of the cost of products, and the cost is $\gamma \frac{k{\bar{\tau_{VM}}}^{2}}{2}$. The retailer needs to provide a proportionate share of profit to the low-carbon enterprise, and the proportion is $\left(1-\beta \right)\left(\bar{p_{VM}}-c+\bar{\tau_{VM}}v\right)\bar{q_{VM}}$, where β,γ∈[0,1].

Therefore, with a cost–benefit-sharing contract, the respective profits of the low-carbon enterprise and the retailer are:

(6) $$\bar{\pi_{VMM}}=\left(1-\beta \right)\left(\bar{p_{VM}}-c+\bar{\tau_{VM}}v\right)\bar{q_{VM}}-\left(1-\gamma \right)\frac{k{\bar{\tau_{VM}}}^{2}}{2}$$

(7) $$\bar{\pi_{VMR}}=\beta \left(\bar{p_{VM}}-c+\bar{\tau_{VM}}v\right)\bar{q_{VM}}-\gamma \frac{k{\bar{\tau_{VM}}}^{2}}{2}$$

Combining Equations (6) and (7), we obtain:

(8) $$\bar{p_{VM}^{*}}=\frac{k\left(1-\gamma \right)\left(Q+\alpha c\right)-\alpha v^{2}\left(1-\beta \right)Q}{\alpha \left[2\left(1-\gamma \right)k-\left(1-\beta \right)\alpha v^{2}\right]}\;$$

(9) $$\bar{q_{VM}^{*}}=\frac{k\left(1-\gamma \right)\left(Q-\alpha c\right)}{2\left(1-\gamma \right)k-\left(1-\beta \right)\alpha v^{2}}$$

(10) $$\bar{\tau_{VM}^{*}}=\frac{v\left(1-\beta \right)\left(Q-\alpha c\right)}{2\left(1-\gamma \right)k-\left(1-\beta \right)\alpha v^{2}}$$

(11) $$\bar{\pi_{VMM}^{*}}=\frac{k\left(1-\beta \right)\left(1-\gamma \right){\left(Q-\alpha c\right)}^{2}}{2\alpha \left[2\left(1-\gamma \right)k-\left(1-\beta \right)\alpha v^{2}\right]}$$

(12) $$\bar{\pi_{VMR}^{*}}=k{\left(Q-\alpha c\right)}^{2}\frac{2\beta k{\left(1-\gamma \right)}^{2}-\alpha \gamma v^{2}{\left(1-\beta \right)}^{2}}{2\alpha {\left[2\left(1-\gamma \right)k-\left(1-\beta \right)\alpha v^{2}\right]}^{2}}$$

We derive Proposition 9 by analyzing Equations (8)–(12) and $\overset{*}{p_{VC}},\;\overset{*}{q_{VC}},\;\overset{*}{\tau_{VC}},\overset{*}{\pi_{VC}}$ in situations of centralized decision making.

**Proposition** **9.** When $\bar{w_{VM}{}^{*}}=\frac{2kc-Qv^{2}}{2k-\alpha v^{2}},\;\beta =\gamma$, LCSC coordination can be achieved through cost–benefit-sharing contracts when the government subsidizes recipients based on carbon emission reductions levels, where $\beta \in \left[\frac{2k\left(2k-\alpha v^{2}\right)}{\alpha {\left(4k-\alpha v^{2}\right)}^{2}},\frac{2k}{4k-\alpha v^{2}}\right]$. Please refer to Appendix A for the specific certification process.

## 4. Numerical Analysis

To further analyze the influences of government subsidies and carbon reduction costs on the optimal solution, this paper uses electric vehicles as a test case for numerical analysis. The subsidy model based on the carbon emission level makes the US automobile fuel industry and the electric power industry’s welfare gains 1% and 36% [58]. In 2020, the Chinese government’s subsidy strategy for electric and plug-in hybrid vehicles was (1) a subsidy of CNY 22,500 for each vehicle with a pure electric range of at least 400 km; (2) a subsidy of CNY 16,200 for each vehicle with a pure electric range between 250 km and 400 km; (3) a subsidy of CNY 8500 for each plug-in hybrid car with a range of no less than 50 km [61,62]. According to [63], after processing the data, it is known that the potential market demand for a certain electric vehicle is 20,000, the unit retail price of consumers is 3, and the unit production cost is 500. Furthermore, according to the maximum and minimum government subsidies for certain electric vehicles, it can be seen that the variation range of the subsidy amount is (80,240). In addition, the variation of the cost coefficient of corporate carbon emission reduction technology is (1,1.5).

### 4.1. The Influences of ν and k on LCP Wholesale Price per Unit

**Figure 2:**
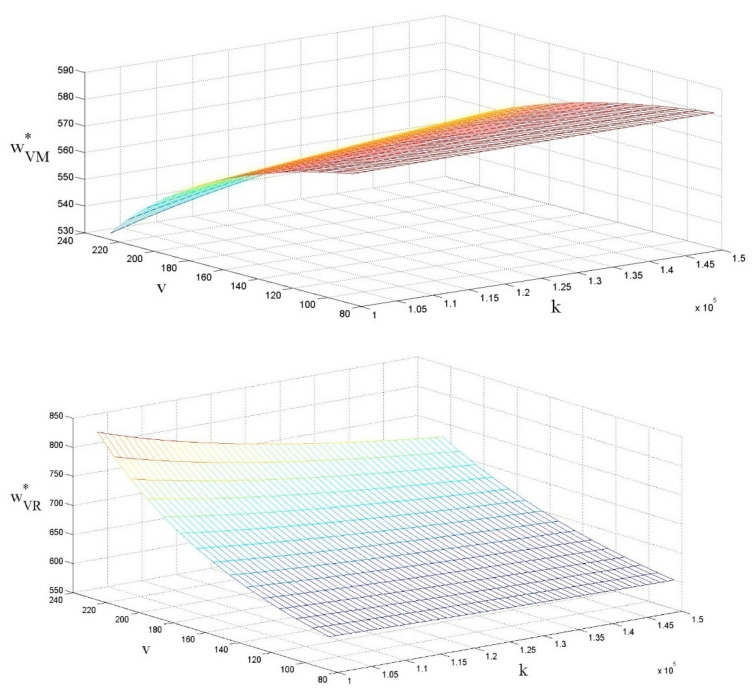
The influences of ν and *k* on LCP wholesale price per unit.

As shown in Figure 2, the influences on the LCP wholesale price per unit depend on whether the government subsidizes low-carbon enterprises or retailers. When the government subsidizes the low-carbon enterprise, the wholesale price per unit of low-carbon product is negatively related to the amount of the government subsidy, which is similar to the findings of [59,60]. This is mainly because government subsidies are issued based on the LCP sales volume, and low-carbon enterprises are incentivized to increase sales by reducing the LCP wholesale price per unit, which indirectly reduces the LCP retail price per unit. On the other hand, when the government subsidizes retailers, low-carbon enterprises tend to increase the wholesale price per unit to receive part of the subsidies, which means that higher government subsidies lead to a higher LCP wholesale price per unit.

In terms of the cost of carbon emission reductions, in contrast to [59,60], when the government subsidizes low-carbon enterprises, the LCP wholesale price per unit is positively correlated with the unit cost factor of carbon emission reduction, and when the government subsidizes retailers, the LCP wholesale price per unit is negatively correlated with the cost factor. This is because, as the recipient of subsidies, low-carbon enterprises choose to obtain more government subsidies by reducing the LCP wholesale price per unit. However, if the carbon emission reduction cost factor becomes high, the low-carbon enterprise will choose to increase the wholesale price per unit of low-carbon products, transferring part of the cost of carbon emission reduction to the retailer. However, when the government is subsidizing, the decrease in the LCP wholesale price per unit should be larger than the increase in the LCP wholesale price per unit due to the increasing cost factors of carbon emission reductions. This means that when the government subsidizes the low-carbon enterprise based on the level of carbon emission reduction, overall, it helps to reduce the LCP wholesale price per unit. In contrast to [59], when the government subsidizes the retailer, the low-carbon enterprise will increase the LCP wholesale price per unit to acquire part of the subsidies from the retailer. When the cost factors of carbon emission reduction increase, the low-carbon enterprise generally chooses to increase the LCP wholesale price per unit to compensate for the increased cost. However, the low-carbon enterprise chooses to reduce the LCP wholesale price per unit to receive more subsidies if the government subsidizes based on the sales volume. According to the above analysis, we can infer the following:

Corollary:

$$\frac{\partial w_{VM}^{*}}{\partial v}<0,\;\frac{\partial w_{VR}^{*}}{\partial v}>0,\;\frac{\partial w_{VM}^{*}}{\partial k}>0,\;\frac{\partial w_{VR}^{*}}{\partial k}<0$$

### 4.2. The Influences of ν and k on the Carbon Emission Reduction Efforts per Unit

**Figure 3:**
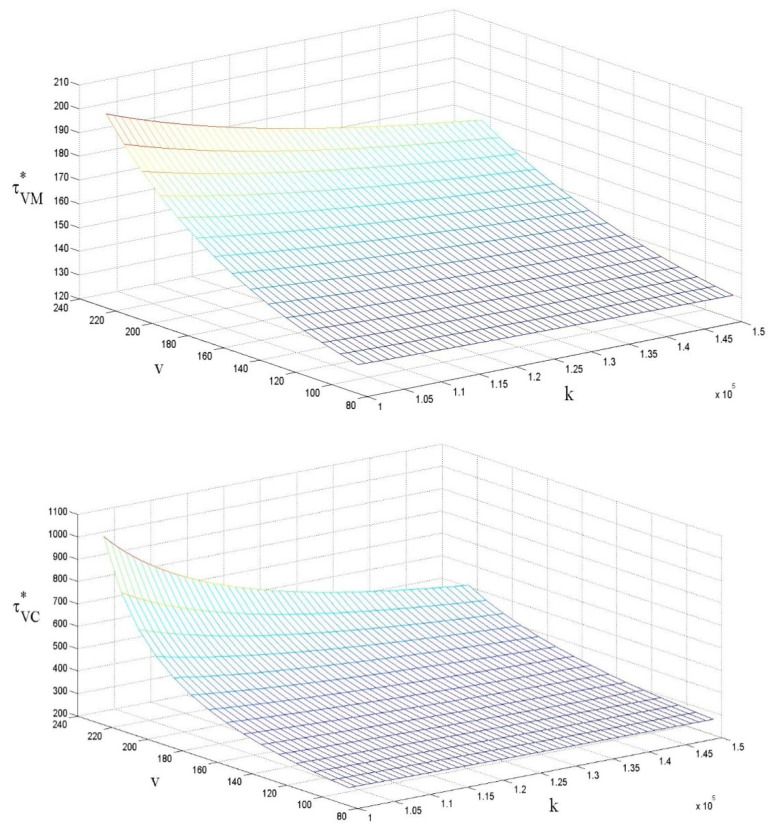
The influences of ν and *k* on the carbon emission reduction efforts per unit.

In contrast to the current literature, Figure 3 shows that the effect of government subsidies on the carbon emission reduction efforts per unit is positive, and the effect of government subsidies on the cost is negative. Moreover, the trend of carbon reduction efforts remains consistent when the government chooses to subsidize different recipients. For the same value of subsidies, the carbon emission reduction efforts per unit in centralized decision making are always greater than in decentralized decision making, and the influence of the cost factor on the reduction effort is more significant in the context of centralized decision making. Therefore, this paper further analyzes the supply chain coordination strategy so that the carbon emission reduction level in the context of decentralized decision making is similar to that obtained in centralized decision making. From the above analysis, we can infer that:

Corollary:

$$\frac{\partial \tau_{VM}^{*}}{\partial v}>0,\;\frac{\partial \tau_{VR}^{*}}{\partial v}>0,\;\frac{\partial \tau_{VM}^{*}}{\partial k}<0,\;\frac{\partial \tau_{VR}^{*}}{\partial k}<0$$

### 4.3. The Influences of ν and k on the Retail Price per Unit

**Figure 4:**
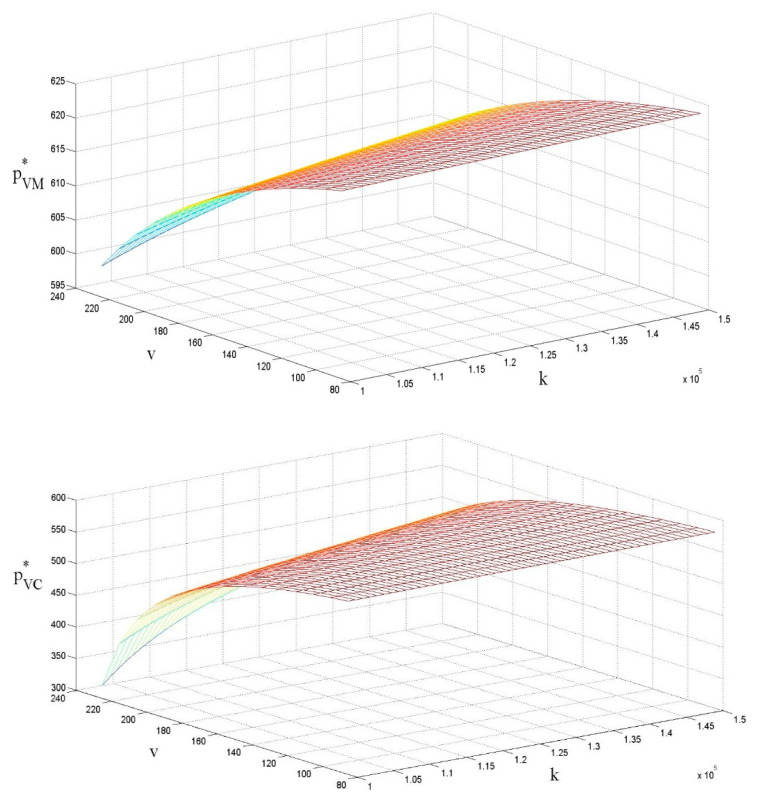
The influences of ν and *k* on the retail price per unit.

Similar to [21], Figure 4 shows that government subsidies significantly reduce the LCP retail price per unit. When the government grants subsidies to the low-carbon enterprise, the low-carbon enterprise reduces the LCP wholesale price per unit, which leads to an indirect reduction in the LCP retail price per unit. When the government grants subsidies to the retailer, the retailer directly reduces the retail price per unit. Given the same value of government subsidies, the LCP retail price per unit in situations of centralized decision making is lower than that in decentralized decision making, but the difference between decision-making models is not significant.

In contrast to [21], the retail price per unit is positively related to the cost factor of carbon emission reductions because the increase in the cost factor results in a rise in the cost of carbon emission reduction. By setting a higher retail price per unit, the retailer is able to transfer part of the cost to consumers. However, the impact of the cost factor on the retail price per unit is not significant. Based on the above analysis, we can infer that:

Corollary:

$$\frac{\partial p_{VM}^{*}}{\partial v}<0,\;\frac{\partial p_{VC}^{*}}{\partial v}<0,\;\frac{\partial p_{VM}^{*}}{\partial k}>0,\;\frac{\partial p_{VC}^{*}}{\partial k}>0\;$$

### 4.4. The Influences of ν and k on the Sales Volume

**Figure 5:**
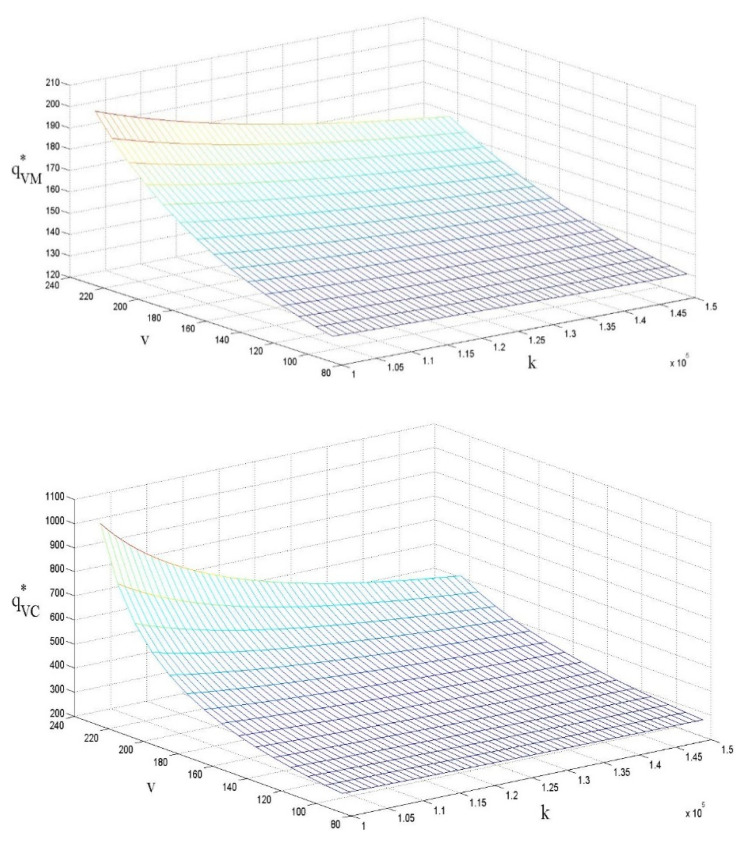
The influences of ν and *k* on the sales volume of the products.

Similar to [21], Figure 5 indicates that the adoption of subsidies by the government could help to improve the sales volume of low-carbon products. Referring back to Figure 3, we argue that government subsidies can help to reduce the LCP retail price per unit, which directly promotes an increase in the sales volume. The decision-making model also has significant impacts on the sales volume of the products. Specifically, when decision making is centralized, the LCP sales volume is significantly higher than when decision making is decentralized. The negative impact of the cost factor on the LCP retail price per unit, on the other hand, is not significant. Based on this, we infer that:

Corollary:

$$\frac{\partial q_{VM}^{*}}{\partial v}>0,\;\frac{\partial q_{VC}^{*}}{\partial v}>0,\;\frac{\partial q_{VM}^{*}}{\partial k}<0,\;\frac{\partial q_{VC}^{*}}{\partial k}<0$$

### 4.5. The Influences of ν and k on Profit

**Figure 6:**
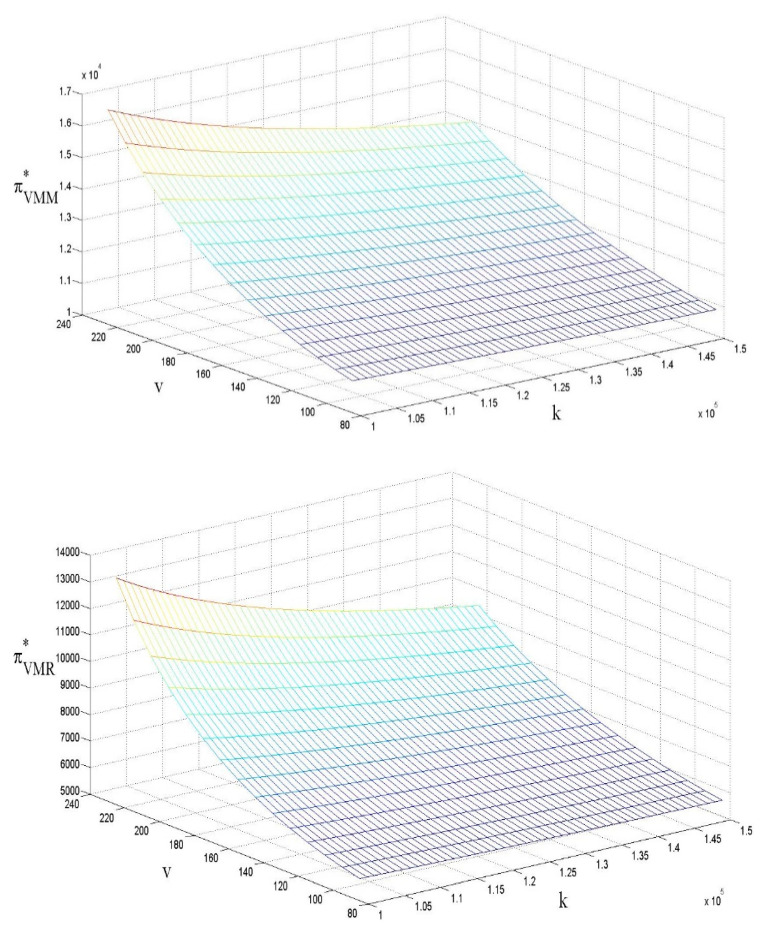
The influences of ν and *k* on profit.

Similar to [54,56], Figure 6 indicates that the adoption of subsidies by the government could increase both the revenue of the low-carbon enterprise and the retailer. Although government subsidies reduce the LCP retail price per unit and result in an initial reduction in revenue, the increase in sales volume induced by the subsidies compensates for the reduction and ultimately increases the revenue. In terms of cost factors, the revenue of both low-carbon enterprises and retailers is negatively related to cost factors. We infer that:

Corollary:

$$\frac{\partial \pi_{VM}^{*}}{\partial v}>0,\;\frac{\partial \pi_{VC}^{*}}{\partial v}>0,\;\frac{\partial \pi_{VM}^{*}}{\partial k}<0,\;\frac{\partial \pi_{VC}^{*}}{\partial k}<0$$

## 5. Conclusions

To improve low-carbon technology, the government shifted its strategy from subsidizing low-carbon products to low-carbon technology. In order to analyze the impact of the government subsidy strategy on the LCSC, a game theory model between a low-carbon enterprise and a retailer was constructed. Based on the game model, we analyzed the impact of government subsidies based on carbon emission reduction levels on the wholesale price, retail price, sales volume, and revenue of low-carbon products per unit. As a result of the analysis, there are three main conclusions and some suggestions.

(1) The main findings of the paper are that a government subsidy strategy based on carbon emission reduction levels can effectively drive low-carbon enterprises to further reduce the carbon emissions. The government’s choice of subsidy has the same effect on the LCP retail price per unit, the sales volume, and the revenue of low-carbon products per unit. When the government subsidizes the retailer, the low-carbon product wholesale price per unit is the highest. That is, low-carbon enterprises use up part of the government subsidies by increasing the wholesale price of low-carbon products.
(2) The retail price of low-carbon products per unit is lower than the retail price of low-carbon products in the context of decentralized decision making, but the sales volume and revenue of low-carbon products are greater in the centralized decision-making. The cost–benefit-sharing contract could enable the decentralized decision model to achieve the same level of profit as the centralized decision model.
(3) The above findings indicate that subsidizing low-carbon enterprises or retailers has the same effect on the low-carbon retail price per unit. Government subsidies based on carbon emission reduction levels can have positive influences on low-carbon enterprises. Government subsidies can cover a part of the carbon emission reduction costs, and the amount of subsidy granted to the low-carbon enterprise is positively related to its carbon emission reduction levels. Overall, a modest increase in government subsidies tied to carbon emission reduction levels can improve the competitiveness and innovativeness of low-carbon enterprises.

The impact of government subsidies on the LCSC in different decision-making contexts was analyzed based on carbon emission reduction levels. The analysis offers some managerial insights for policy makers when they implement sustainability-related initiatives. The findings in this study highlight the necessity of further research. For instance, what is the impact of consumer preferences regarding carbon emission reduction levels on the LCSC? What is the impact of government subsidies on market competition between LCPs and regular products? What is the optimal value of government subsidies? Although government subsidies can effectively drive low-carbon companies to upgrade their carbon emission reduction levels, they will also cause some polluting companies to enter the industry, which will affect the pace of carbon emission reduction and carbon neutralization. In addition, with the improvement of carbon emission reduction technology, some countries have gradually changed the government subsidy-based policy to adopt a carbon tax-based policy. This is also a key aspect that can be expanded in future research.

## Figures and Tables

**Table 1:** The meanings of parameters, variables and functions.

Symbols	Meaning
VC	The situations of the decentralized decision model
c	Production costs of low-carbon products (LCP) per unit
VM	Government subsidies to the low-carbon enterprise in situations of decentralized decision making
VR	Government subsidies to the retailer in situations of decentralized decision making
k	Cost factor for carbon emission reduction efforts of LCP per unit
Q	Potential demand in the LCP market when the LCP price per unit is zero
α	Consumer sensitivity to the LCP retail price per unit
v	The LCP subsidy per unit
$w_{i}$	In case i, the LCP wholesale price per unit, where i∈{VM,VR}
$p_{i}$	In case i, the retail price of LCP per unit, where i∈{VM,VR,VC}
$q_{i}$	In case i, sales volumes of LCP, where i∈{VM,VR,VC}
$\tau_{i}$	In case i, the unit levels of carbon emission reduction effort, where i∈{VM,VR,VC}
$\pi_{iM}$	In case i, the low-carbon enterprise’s profit, where i∈{VM,VR,VC}
$\pi_{iR}$	In case i, the retailer’s profit, where i∈{VM,VR,VC}
$\pi_{VC}$	Profits of the LCSC in situations of centralized decision making
$\bar{\pi_{VC}}$	Total profits of the LCSC when the government subsidizes the low-carbon enterprise
$\bar{\pi_{VR}}$	Total profits of the LCSC when the government subsidizes the retailer
$\bar{w_{VM}}$	The LCP wholesale price per unit under a cost–benefit-sharing contract when the government subsidizes the low-carbon enterprise
$\bar{p_{VM}}$	The LCP retail price per unit under a cost–benefit-sharing contract when the government subsidizes the low-carbon enterprise
$\bar{q_{VM}}$	Sales volume of LCP under a cost–benefit-sharing contract when the government subsidizes the low-carbon enterprise
$\bar{\tau_{VM}}$	Carbon emission reduction efforts under a cost–benefit-sharing contract when the government subsidizes the low-carbon enterprise
$\bar{\pi_{VMM}}$	Low-carbon enterprise’s profit under a cost–benefit-sharing contract when the government subsidizes the low-carbon enterprise
$\bar{\pi_{VMR}}$	Retailer’s profit in situations of a cost–benefit-sharing contract when the government subsidizes the low-carbon enterprise

**Table 2:** Optimal solution.

Symbols	i=VM	i=VR	i=VC
$w_{i}^{*}$	$\frac{2k\left(Q+\alpha c\right)-\alpha Qv^{2}}{\alpha \left(4k-\alpha v^{2}\right)}$	$\frac{2k\left(Q+\alpha c\right)-\alpha^{2}v^{2}c}{\alpha \left(4k-\alpha v^{2}\right)}$	——
$\tau_{i}^{*}$	$\frac{v\left(Q-\alpha c\right)}{4k-\alpha v^{2}}$		$\frac{v\left(Q-\alpha c\right)}{2k-\alpha v^{2}}$
$p_{i}^{*}$	$\frac{k\left(3Q+\alpha c\right)-\alpha Qv^{2}}{\alpha \left(4k-\alpha v^{2}\right)}$		$\frac{k\left(Q+\alpha c\right)-\alpha Qv^{2}}{\alpha \left(2k-\alpha v^{2}\right)}$
$q_{i}^{*}$	$\frac{k\left(Q-\alpha c\right)}{4k-\alpha v^{2}}$		$\frac{k\left(Q-\alpha c\right)}{2k-\alpha v^{2}}$
$\pi_{iM}^{*}$	$\frac{k{\left(Q-\alpha c\right)}^{2}}{2\alpha \left(4k-\alpha v^{2}\right)}$		——
$\pi_{iR}^{*}$	$\frac{k^{2}{\left(Q-\alpha c\right)}^{2}}{\alpha {\left(4k-\alpha v^{2}\right)}^{2}}$		——
$\pi_{i}^{*}$	——		$\frac{k{\left(Q-\alpha c\right)}^{2}}{2\alpha \left(2k-\alpha v^{2}\right)}$

## Data Availability

The data comes from the official website of the Chinese government and the “Notice on Soliciting Public Opinions on Several Policies for Promoting the Development of New Energy Vehicles in Guangzhou” issued by the Guangzhou Development and Reform Commission.

## Appendix A

**Proof.** The proof of Lemma 1:

Equation (1) Substituting $q_{VM}=Q-\alpha p_{VM}$ into Equation (2) yields:

(A1) $$\pi_{VMR}=\left(p_{VM}-w_{VM}\right)\left(Q-\alpha p_{VM}\right)$$

The first-order and second-order partial derivatives are obtained for Equation (A1) with respect to $\;p_{VM}$, which yields:

(A2) $$\frac{\partial \pi_{VMR}}{\partial p_{VM}}=Q-2\alpha p_{VM}+\alpha w_{VM}$$

(A3) $$\frac{\partial^{2}\pi_{VMR}}{\partial p_{VM}{}^{2}}=-2\alpha$$

From Equation (A3), we obtain $\frac{\partial^{2}\pi_{VMR}}{\partial p_{VM}^{2}}=-2\alpha <0$, so Equation (2) with respect to $\;p_{VM}$ is a concave function. Setting Equation (A2) equal to zero results in $\overset{*}{\;p_{VM}}=\frac{Q-\alpha w_{VM}}{2\alpha }$. Substituting $\overset{*}{\;p_{VM}}$ into $q_{VM}=Q-\alpha p_{VM}$ results in $\overset{*}{q_{VM}}=\frac{Q-\alpha w_{VM}}{2}$, and substitutin $\overset{*}{q_{VM}}=\frac{Q-\alpha w_{VM}}{2}$ into Equation (A1) results in:

(A4) $$\pi_{VMM}=\frac{\left(w_{VM}-c+\tau_{VM}v\right)\left(Q-\alpha w_{VM}\right)-k\tau_{VM}^{2}}{2}$$

The first-order and second-order partial derivatives of Equation (A4) with respect to $w_{VM}$ and $\;\tau_{VM}$, respectively, are obtained as follows:

(A5) $$\frac{\partial \pi_{VMM}}{\partial w_{VM}}=\frac{Q-2\alpha w_{VM}+\alpha c-\alpha \tau_{VM}v}{2}$$

(A6) $$\frac{\partial \pi_{VMM}}{\partial \tau_{VM}}=\frac{Qv-\alpha w_{VM}v-2k\tau_{VM}}{2}$$

(A7) $$\frac{\partial^{2}\pi_{VMM}}{\partial \tau_{VM}\partial w_{VM}}=\frac{\partial^{2}\pi_{VMM}}{\partial w_{VM}\partial \tau_{VM}}=-\frac{\alpha v}{2}$$

(A8) $$\frac{\partial^{2}\pi_{VMM}}{\partial w_{VM}{}^{2}}=-\alpha ,\;\frac{\partial^{2}\pi_{VMM}}{\partial \tau_{VM}{}^{2}}=-k\;$$

From Equations (A5)–(A8), we obtain the Hessian matrix of Equation (A1) with respect to $w_{VM},\;\tau_{VM}$:

$$H=\left[\begin{array}{cc} -\alpha & -\frac{\alpha v}{2}\\ -\frac{\alpha v}{2} & -k \end{array}\right]$$

The determinant of the Hessian matrix is $\left|H\right|=\alpha \frac{4\alpha -\alpha v^{2}}{4}>0$, and the first-order principal sub-equation −α<0, so Equation (1) with respect to $w_{VM},\;\tau_{VM}$ is a concave function. Thus, (i) is proved, and similarly, (ii) and (iii) are proved. □

**Proof.** The proof of Proposition 3:

$$w_{VM}^{*}-w_{VR}^{*}=-\alpha v^{2}\frac{Q-\alpha c}{\alpha \left(4k-\alpha v^{2}\right)}<0,\;i.e.,\;\overset{*}{w_{VM}}<\overset{*}{w_{VR}}$$

□

**Proof.** The proof of Proposition 4:

(i) $\frac{\partial w_{VM}^{*}}{\partial v}=-\frac{4kv\left(Q-\alpha c\right)}{{\left(4k-\alpha v^{2}\right)}^{2}}<0$,$\frac{\partial w_{VR}^{*}}{\partial v}=\frac{4kv\left(Q-\alpha c\right)}{{\left(4k-\alpha v^{2}\right)}^{2}}>0$;
(ii) $\frac{\partial p_{VM}^{*}}{\partial v}=\frac{\partial p_{VR}^{*}}{\partial v}=\frac{-2kv\left(Q-\alpha c\right)}{{\left(4k-\alpha v^{2}\right)}^{2}}<0,\;\frac{\partial p_{VC}^{*}}{\partial v}=\frac{-2kv\left(Q-\alpha c\right)}{{\left(2k-\alpha v^{2}\right)}^{2}}<0$;
(iii) $\frac{\partial q_{VM}^{*}}{\partial v}=\frac{\partial q_{VR}^{*}}{\partial v}=\frac{2\alpha kv\left(Q-\alpha c\right)}{{\left(4k-\alpha v^{2}\right)}^{2}}>0,\;\frac{\partial q_{VC}^{*}}{\partial v}=\frac{2\alpha kv\left(Q-\alpha c\right)}{{\left(2k-\alpha v^{2}\right)}^{2}}>0$. □

**Proof.** The proof of Proposition 5:

$$\frac{\partial \tau_{VM}^{*}}{\partial v}=\frac{\partial \tau_{VR}^{*}}{\partial v}=\frac{2\alpha \left(Q-\alpha c\right)v^{2}}{{\left(4k-\alpha v^{2}\right)}^{2}}>0,\;\frac{\partial \tau_{VC}^{*}}{\partial v}=\frac{2\alpha \left(Q-\alpha c\right)v^{2}}{{\left(2k-\alpha v^{2}\right)}^{2}}>0$$

□

**Proof.** The proof of Proposition 6:

(i) $\frac{\partial \pi_{VMM}^{*}}{\partial v}=\frac{\partial \pi_{VRM}^{*}}{\partial v}=\frac{kv{\left(Q-\alpha c\right)}^{2}}{{\left(4k-\alpha v^{2}\right)}^{2}}>0$;
(ii) $\frac{\partial \pi_{VMR}^{*}}{\partial v}=\frac{\partial \pi_{VRR}^{*}}{\partial v}=\frac{k^{2}{\left(Q-\alpha c\right)}^{2}v}{{\left(4k-\alpha v^{2}\right)}^{3}}>0$;
(iii) $\frac{\partial \pi_{VC}^{*}}{\partial v}=\frac{kv{\left(Q-\alpha c\right)}^{2}}{{\left(2k-\alpha v^{2}\right)}^{2}}>0$. □

**Proof.** The proof of Proposition 7:

(i) $p_{VM}^{*}-p_{VC}^{*}=p_{VR}^{*}-p_{VC}^{*}=\frac{2k^{2}\left(Q-\alpha c\right)}{\alpha \left(2k-\alpha v^{2}\right)\left(4k-\alpha v^{2}\right)}>0$;
(ii) $q_{VC}^{*}-q_{VM}^{*}=q_{VC}^{*}-q_{VR}^{*}=\frac{2k^{2}\left(Q-\alpha c\right)}{\left(2k-\alpha v^{2}\right)\left(4k-\alpha v^{2}\right)}>0$;
(iii) $\tau_{VC}^{*}-\tau_{VM}^{*}=\tau_{VC}^{*}-\tau_{VR}^{*}=\frac{2kv\left(Q-\alpha c\right)}{\left(2k-\alpha v^{2}\right)\left(4k-\alpha v^{2}\right)}>0$. □

**Proof.** The proof of Proposition 8:

$$\pi_{VC}^{*}-\pi_{VM}^{*}=\pi_{VC}^{*}-\pi_{VR}^{*}=\frac{4k^{3}{\left(Q-\alpha c\right)}^{2}}{2\alpha \left(2k-\alpha v^{2}\right){\left(4k-\alpha v^{2}\right)}^{2}}□$$

□

**Proof.** The proof of Proposition 9:

When β=γ,

$\bar{w_{VM}{}^{*}}=\frac{2kc-Qv^{2}}{2k-\alpha v^{2}},\;\bar{p_{VM}^{*}}=p_{VM}^{*},\;\bar{q_{VM}^{*}}=q_{VM}^{*},\;\bar{\tau_{VM}^{*}}=\tau_{VM}^{*}$

$\bar{\pi_{VMM}^{*}}=\frac{k\left(1-\beta \right){\left(Q-\alpha c\right)}^{2}}{2\alpha \left(2k-\alpha v^{2}\right)},\;\bar{\pi_{VMR}^{*}}=\frac{\beta k{\left(Q-\alpha c\right)}^{2}}{2\alpha \left(2k-\alpha v^{2}\right)}$

So,

$\bar{\pi_{VMM}^{*}}\geq \pi_{VMM}^{*}\Leftrightarrow \beta \leq \frac{2k}{4k-\alpha v^{2}}$,

$\bar{\pi_{VMR}^{*}}\geq \pi_{VMR}^{*}\Leftrightarrow \frac{2k\left(2k-\alpha v^{2}\right)}{\alpha {\left(4k-\alpha v^{2}\right)}^{2}}\leq \beta$, i.e., $\beta \in \left[\frac{2k\left(2k-\alpha v^{2}\right)}{\alpha {\left(4k-\alpha v^{2}\right)}^{2}},\frac{2k}{4k-\alpha v^{2}}\right]$.

Therefore, this paper argues that, when the government subsidizes the retailer, as long as $\bar{w_{VR}{}^{*}}=c$, and the proportion of cost–benefit-sharing remains unchanged, the coordination of the LCSC can be achieved. □
